# Phenotypes of Hereditary Diseases Associated With Rauch–Steindl Syndrome

**DOI:** 10.1155/humu/1637135

**Published:** 2026-09-03

**Authors:** Yi Kong, Yousheng Wang, Xingwang Wang, Li Guo, Jianhong Chen, Yan Ji, Jiaolan Liu, Wanfang Xu, Weiling Du, Haiyan Wang, Yan Zhang

**Affiliations:** ^1^ Graduate School, Guangzhou Medical University, Guangzhou, China, gzhmu.edu.cn; ^2^ Department of Medicine, Hangzhou Juno Genomics Inc., Hangzhou, China, duhs.edu.pk; ^3^ Department of Primary Care Collaboration, Guangdong Women and Children Hospital, Guangzhou, China, e3861.com; ^4^ Medical Genetic Center, Guangdong Women and Children Hospital, Guangzhou, China, e3861.com; ^5^ Medical Genetic Center, Women and Children′s Hospital, Southern University of Science and Technology, Guangzhou, China, sustc.edu.cn; ^6^ Guangzhou Key Laboratory of Prenatal Screening and Prenatal Diagnosis, Guangzhou, China; ^7^ Maternal and Children Metabolic-Genetic Key Laboratory, Guangzhou, China; ^8^ Department of Obstetrics, Huizhou First Maternal and Child Health Care Hospital, Huizhou, China; ^9^ Department of Obstetrics, Huizhou Municipal Central Hospital, Huizhou, China, hzch.gd.cn; ^10^ Department of Obstetrics, Chang’An Hospital of Dongguan, Dongguan, China; ^11^ Department of Obstetrics, Dongguan Maternal and Child Health Care Hospital, Dongguan, China; ^12^ Department of Obstetrics, Huizhou First People′s Hospital, Huizhou, China

**Keywords:** *Nsd2*, phenotypes, Rauch–Steindl syndrome

## Abstract

**Purpose:**

Prenatal phenotypic manifestations of genetic disorders associated with *NSD2* variants remain poorly characterized. This study presents our institutional experience with the prenatal diagnosis of *NSD2*‐associated genetic disorders, specifically Rauch–Steindl syndrome (RAUST), aiming to improve understanding of both the molecular and clinical features of RAUST.

**Methods:**

We performed a retrospective analysis of six fetuses and one adult diagnosed with RAUST at our institution and thoroughly reviewed the prenatal ultrasound reports of six fetuses. Prenatal and postnatal phenotypes of RAUST cases were summarized alongside findings from previously published literature. Correlations between NSD2 variant locations, variant types, and phenotypes were analyzed. Additionally, protein modeling was used to visualize structural changes in NSD2 protein before and after C‐terminal variants. We integrated single‐cell transcriptomic and gene expression data from multiple public databases to investigate spatiotemporal expression patterns of *NSD2* during human fetal development.

**Results:**

Fetal growth restriction (FGR) was the most prevalent prenatal manifestation in RAUST fetuses, followed by microcephaly. Bilateral renal hypoplasia emerged as a novel prenatal ultrasonographic feature. Postnatally, speech and motor developmental delays were the most commonly reported phenotypes, followed by physical developmental delays and intellectual disability. Genotype‐phenotype correlation analysis revealed an association between N‐terminal truncating variants in NSD2 and impaired fetal growth parameters. Notably, C‐terminal truncating variants—predicted not to directly impact NSD2 functional domains—also exerted disease‐causing effects.

**Conclusion:**

This study provides a comprehensive analysis of prenatal phenotypes in RAUST cases, enriching the prenatal phenotypic spectrum of the disease and facilitating early diagnosis and clinical management of RAUST. Furthermore, our genotype‐phenotype correlation findings lay a foundational basis for future research into the complex molecular mechanisms underlying *NSD2*‐associated genetic disorders.

## 1. Introduction

Rauch–Steindl syndrome (RAUST) is a rare genetic disorder that was distinguished from Wolf–Hirschhorn syndrome (WHS) in 2018 due to its comparatively milder clinical features [1]. RAUST is characterized by growth retardation, facial deformities, mild skeletal abnormalities, and psychological and behavioral issues [2]. The deficits in language and motor development of affected children are difficult to fully remediate through subsequent rehabilitation, resulting in long‐term adverse effects on future learning and social life.

As with most genetic disorders, RAUST demonstrates substantial clinical heterogeneity [3]. Traditionally, diagnosis is established postnatally through clinical symptoms followed by genetic testing. Comprehensive reports on prenatal ultrasound abnormalities in RAUST cases remain scarce globally. The lack of specific prenatal ultrasound markers for RAUST presents a significant challenge to early diagnosis.

As an autosomal dominant disease, RAUST is caused by *NSD2* gene, which is located on chromosome 4p16.3 and encodes the nuclear receptor‐binding SET domain protein 2 (NSD2). NSD2 consists of four structural domains: PWWP (Proline‐Tryptophan‐Tryptophan‐Proline) (A “reader” that recognizes and binds to methylated histone and DNA), SET (Su (Var)3‐9, Enhancer‐of‐zoster and Trithorax) (A “writer” that catalyzes the transfer of methyl groups to lysine residues on histones), PHD (Plant Homeo Domain) (A “epigenetic reader” that specially recognizes the methylation of histone H3K4)‐type zinc‐finger domains, and the HMG (High Mobility Group) (A architectural element that binds to DNA to induce structural changes like bending and looping) [1]. NSD2 plays a critical role in the methylation of histones, specifically catalyzing the methylation of lysine 36 on histone 3 (H3K36me2) [4]. Its methyltransferase activity is associated with actively transcribed genomic regions during embryonic development [3, 5].

To date, there is a paucity of correlation analyses between *NSD2* variants and their corresponding phenotypes. Among the over 40 identified *NSD2* variants, missense and truncation variants are distributed throughout the gene [6]. Zanoni’s research demonstrated that patients with missense variants exhibited less severe physical developmental delays but more frequent behavioral and psychological issues compared with those with *NSD2* truncation variants [3]. Nevertheless, further investigation into the correlation between genetic variants and phenotypes remains imperative. This study aims to summarize the incidence of phenotypes in fetuses diagnosed with RAUST. Detailed fetal ultrasound abnormalities and *NSD2* variants were analyzed. Cases reported in relevant literature were also included and analyzed, thus providing new insights and perspectives for the early diagnosis of RAUST.

## 2. Materials and Methods

### 2.1. Study Design and Patient Selection

This retrospective study included six fetuses and one adult diagnosed with RAUST between January 2021 and March 2025 at Guangdong Women and Children Hospital. Six fetuses were found to have fetuses with small for gestational age (SGA) or fetal growth restriction (FGR) on prenatal ultrasound scanning. After going through the routine prenatal screening and prenatal diagnosis processes, factors such as aneuploidy and infection were excluded. Through amniotic fluid whole exome sequencing (WES) variations in the *NSD2* gene were found.

Informed consent was obtained from all study participants through signed consent forms for prenatal diagnostic procedures and genetic testing. The study protocol was approved by the Ethics Committee of Guangdong Women and Children Hospital.

### 2.2. WES Detection and Result Analysis

#### 2.2.1. WES Data Acquisition

Targeted genomic enrichment was performed using the xGen Exome Research Panel v1.0 (IDT, United States), followed by sequencing on the Illumina NovaSeq 6000 platform to generate 150 bp paired‐end reads.

#### 2.2.2. WES Data Analysis

Raw reads were first aligned to the human reference genome hg19/GRCh37 using BWA. Variant detection and base quality recalibration were performed with GATK’s HaplotypeCaller. Functional annotation of variants was conducted using VEP and Annovar. To characterize the population genetics of the variants, each site was compared against large‐scale databases such as gnomAD, 1000 Genomes, ESP6500, and ExAC. Additionally, VarCards was employed to assess the impact of variants on protein coding and function. Final variant classifications adhered strictly to the guidelines published jointly by the American College of Medical Genetics and Genomics (ACMG) and the Association for Molecular Pathology (AMP) in 2015.

### 2.3. Data Collection and Analysis

The ultrasonographic findings of the six fetuses were documented in detail, including various anthropometric parameters such as biparietal diameter (BPD), head circumference (HC), abdominal circumference (AC), and femur length (FL). Estimated fetal weight (EFW) and the standard deviation (SD) of each measurement trajectory were calculated using Hadlock3 and individualized formulas [7, 8]. Subsequently, growth charts for each parameter were constructed across gestational weeks to monitor fetal growth and development. Additionally, letter‐labeled comparative box plots of the SD values for each trajectory were generated to assess the distribution of deviations from normative values.

The analyses were performed using the R software package (R4.3.1) “agricolae” via CNSknowall (http://cnsknowall.com/index.html#/HomePage).

### 2.4. Literature Review

We conducted a comprehensive review of RAUST‐related literature published between October 2018 and March 2025. PubMed and OMIM databases were searched using the keywords “Rauch–Steindl syndrome”, “*NSD2*”, and “*WHSC1*”. The inclusion criteria required a diagnosis of RAUST through prenatal or postnatal genetic testing, along with documentation of the corresponding clinical phenotype. Excluding polygenic variations, 10 papers were included. Each eligible case underwent detailed statistical analysis, encompassing the prenatal and postnatal phenotypes of the patients and their genotypes.

### 2.5. Integration and Visualization of Data

By integrating data from existing literature with our dataset, we constructed two bar charts to systematically analyze the incidence of abnormal prenatal ultrasound findings and postpartum phenotypes in RAUST cases. Furthermore, we developed a sample distribution map to analyze the phenotypes of patients based on the position of NSD2 variant along the peptide chain (from the N‐terminus to the C‐terminus). We also created a donut chart to analyze the relationship between the types of NSD2 variants (missense or truncation) and their corresponding phenotypes. Fisher’s exact test was conducted to determine the *p* values for the associations between variant site (domain vs. nondomain), variant type (missense vs. truncating), and the corresponding clinical phenotypes.

### 2.6. Protein Structure Analysis

Homology modeling of full‐length wild‐type human NSD2 and its truncation mutants (p.Trp1358∗, p.Pro1343Glnfs∗49, and p.Glu1344Lysfs∗49) was performed using the Swiss‐Model online tool [9]. A sequence identity above 40% generally ensures high credibility of homologous structural prediction [10–12]. The Global Model Quality Estimate (GMQE) is a comprehensive quality metric trained to predict the lDDT score of structural models, ranging from 0 to 1, with higher values indicating better model reliability [13, 14]. The mouse Nsd2 homolog (UniProt Q8BVE8.1; AlphaFold Database ID: AF‐Q8BVE8‐F1) was selected as the optimal template, with ∼91% sequence identity to human NSD2, 99% full‐length query coverage, and a favorable GMQE score of 0.66. All three‐dimensional structure models were constructed based on the reference transcript NM_133330.2.

### 2.7. Database‐Based *NSD2* Spatiotemporal Expression in Embryonic Development

We integrated single‐cell transcriptomic and gene expression data from various publicly available databases to analyze the spatiotemporal expression characteristics of *NSD2* during human fetal development. The specific data sources include the following: single‐cell RNA‐seq maps and *NSD2* expression data from nine developmental tissues during pregnancy in the Human Developmental Cell Atlas (HDCA, https://developmental.cellatlas.io/); scatter plots of *NSD2*‐associated circular RNA expression in human fetal tissues from the National Center for Biotechnology Information (NCBI, https://www.ncbi.nlm.nih.gov/gene/) [15], quantified in RPKM (Reads Per Kilobase of Exon model per Million Mapped Reads) for six tissues during weeks 8 to 22; *NSD2* expression data in various brain regions from early embryonic stages to post‐birth from the Human Brain Transcriptome database (HBT, https://hbatlas.org/); and *NSD2* expression distribution data in human kidneys at 17 weeks of gestation provided by the Renal Regeneration Research Alliance (RBK, https://www.atlas-d2k.org/).

## 3. Results

The enrolled six pregnant women had an average age of 31.3 ± 4.4 years, and the gestational age at the diagnosis of fetal growth retardation was 20.6 ± 2.8 weeks (Table 1). Several indicators for evaluating fetal development, such as HC (0.429 SD to −4.209 SD), BPD (−0.223 SD to −3.789 SD), AC (0.594 SD to −4.227 SD), and FL (1.031 SD to −3.321 SD), were all significantly behind the gestation of the affected fetuses.

**Table 1:** Detailed data for the six fetal cases.

Case	Mother age (years)	FGR	HC and BPD < −2SD	Detection week of FGR	Other abnormalities	Variant (NM_133330.3)	ACMG evaluation	ACMG criteria	Outcome
1	36	(+)	(+)	17 + 5	ARSA	c.4073G>A, p.(Trp 1358∗) c.1041_1042dup	VUS	PS2	Childbirth
2	24	(+)	(+)	17 + 3	ARSA, VSD, bilateral renal hypoplasia	p.(Tyr348Serfs∗13)	P	PVS1,PS2, PM2	TOP
3	30	(+)	(+)	19 + 6	(—)	c.1676_1679del p.(Arg559Thrfs∗38)	P	PVS1, PS2, PM2	TOP
4	35	(+)	(+)	23 + 6	(—)	NC_000004.11:g.1725545‐2000228del 275Kb del	P	PVS1, PS2, PM2	TOP
5	33	(+)	(+)	23 + 4	Bilateral renal hypoplasia, nonvisualization of the gallbladder	c.2470C>T p.(Arg824∗)	P	PVS1,PS2, PM2	TOP
6	30	(+)	(+)	21	(—)	c.2990del p.(Asn997IlefsTer37)	LP	PVS1, PM2	TOP

*Note:* The genomic references for cases in our institution are all based on hg19. VUS: Variants of Unknown Significance; P: pathogenic; LP: likely pathogenic.

Abbreviations: ARSA: aberrant right subclavian artery; BPD: biparietal diameter; FGR: fetal growth restriction; HC: head circumference; NC: nucleotide complete genomic reference sequence; NM: nucleotide messenger RNA reference sequence; SD: standard deviation; TOP: termination of pregnancy; VSD: ventricular septal Defect.

**Figure 1:**
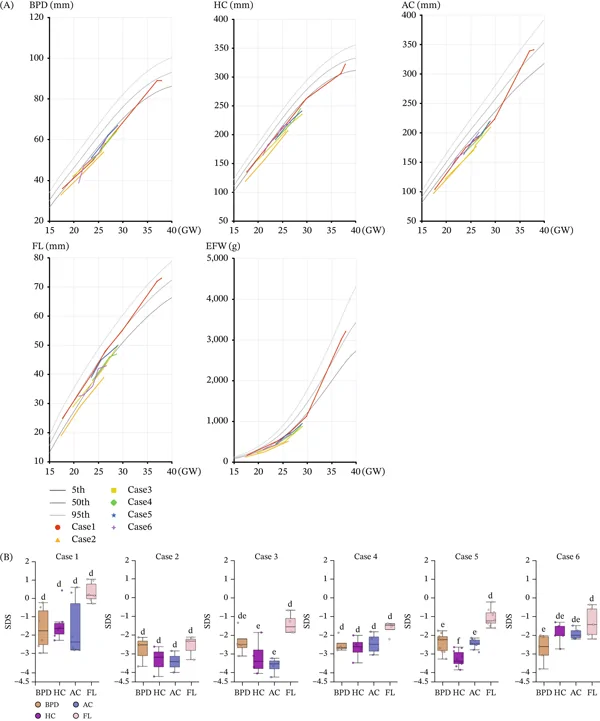
Prenatal ultrasound analysis of growth parameters in six fetuses. (A) Growth chart of six fetuses. The longitudinal growth patterns of six fetuses with *NSD2* variants. (B) Letter‐labeled comparative boxplot (d, e, f, g) for six fetuses. Each boxplot represents a specific growth parameter; distinct letters represent different values, indicating statistically significant differences between groups. Specifically, different letters in d, e, f, and g denote significant differences among groups, while shared letters indicate no significant difference between groups. AC: abdominal circumference; BPD: biparietal diameter; FL: femur length; GW: gestational weeks; HC: head circumference; SD: standard deviation.

Early‐onset FGR, with HC and BPD measurements below the expected range for gestational age, was observed in all six cases. The growth curves (Figure 1A, Supporting Information 1), plotted according to gestational weeks, illustrate the developmental status of the six fetal patients. Except for Case 1, which showed signs of improvement at the end of pregnancy, the other five cases all exhibited persistent lag in growth and development. Specifically, Case 2 manifested a gradually worsening developmental delay. Compared with symmetrical FGR, in the developmental lag related to *NSD2*, the degrees of various measurement indices are not consistent. The reduction in FL was less pronounced but showed statistically significant differences with reductions in HC, AC, and BPD (*p* < 0.05). Additionally, in Case 5, the reduction in HC also exhibited significant differences with reductions in BPD and AC (*p* < 0.05) (Figure 1B).

Besides FGR, bilateral renal anomalies were identified in two cases (Table 1), which have not been previously reported in cases of RAUST.

Five fetuses exhibited de novo variants, while one fetus (Case 6) demonstrated a variant inherited from an affected mother. All six fetuses have truncating variants. As demonstrated in Figure 1B, the severity of FGR or SGA ranked in descending order as follows: Case 2 [p.(Tyr348Serfs∗13)], Case 3 [p.(Arg559Thrfs∗38)], Case 5 [p.(Arg824∗)], Case 6 [p.(Asn997IlefsTer37)], and Case 1 [p.(Trp1358∗)]. This ranking reflects the location of NSD2 truncation in affected cases, demonstrating that the severity of fetal growth parameter impairment escalates as the truncation site progresses toward the N‐terminal region of NSD2 protein. *NSD2* gene is entirely deleted in Case 4, yet FGR is not the most severe.

Five of six pregnant women requested termination of pregnancy following prenatal diagnosis of RAUST. One woman (Case 1) with an *NSD2* variant of uncertain significance chose to carry the fetus to term, resulting in the delivery of a child exhibiting clinical features of physical and motor–verbal developmental delays consistent with RAUST.

### 3.1. Growth Parameters in Enrolled Cases

#### 3.1.1. Case 1

At 17 weeks and 5 days of gestation, routine ultrasound examination indicated FGR, with an AC of 104 mm (−2.76 SD) and an EFW of 172 g (eighth percentile). At 24 weeks of gestation, FGR persisted, with an AC of 175 mm (−2.023 SD) and an EFW of 527 g (5.7th percentile). By 26 weeks and 3 days of gestation, FGR continued, with an AC of 191 mm (−2.765 SD) and an EFW of 729 g (2.5th percentile). Bilateral choroid plexus cysts were identified at 17 weeks and 5 days of gestation and resolved by 24 weeks. BPD below the expected range for gestational age was observed at 24 weeks, measuring 50 mm (−2.928 SD), and persisted at 26 weeks and 3 days, measuring 58 mm (−2.548 SD). Magnetic resonance imaging (MRI) at 29 weeks and 6 days confirmed that the fetal BPD remained below the expected range for gestational age, with no other significant cranial abnormalities detected. HC below the expected range for gestational age was noted at 26 weeks and 3 days, with a measurement of 225 mm (−2.247 SD). Ultrasound examinations at 37 weeks and 37 weeks plus 6 days demonstrated that fetal HC, BPD, AC, FL, and EFW were all within normal ranges.

The infant was delivered via cesarean section at 37 weeks and 6 days of gestation, with Apgar scores of 9‐10‐10, a birth weight of 3.1 kg, and a postpartum blood glucose level of 1.9 mmol/L. After intensive feeding, the retested blood glucose level increased to 3.9 mmol/L, with all other parameters normal. Postnatal cranial MRI showed normal results. By 6 months of age, the infant exhibited decreased muscle tone and lactose intolerance. Her height was 64.8 cm (18th percentile), and her weight was 6 kg (first percentile). At 10 months, the child was unable to roll over or maintain seated stability. By 18 months, the child achieved independent walking, but at 2 years and 3 months of age, verbal expression was limited to single words and fragmented phrases. Her height measured 83.5 cm, placing her at the third percentile, while her weight was 9 kg, corresponding to the 0th percentile. At the most recent follow‐up, the child was 3 years old, could construct sentences consisting of two to three words, used a spoon for eating, performed simple movements, measured 91.7 cm (15th percentile) in height, and weighed 10.6 kg (0th percentile). Meanwhile, the child smiled, rolled over, sat up, stood without assistance, walked independently, used the toilet, and began to speak approximately 2–3 months later than the average toddler. No indications of autism, seizures, distinctive facial features, or skeletal or limb abnormalities were observed. The child exhibited an immunocompromised status and experienced recurrent fevers every 1–3 months, attributed to upper respiratory infections. Additionally, the infant presented a history of gastrointestinal dysfunction, characterized by irregular bowel movements. Her mother had gestational diabetes mellitus during pregnancy, along with moderate anemia.

#### 3.1.2. Case 2

At 17 weeks and 3 days of gestation, ultrasonography revealed FGR. The AC measured 97 mm (−3.422 SD), and the EFW was 131 g (0.1st percentile). Additionally, the BPD (33 mm, −2.144 SD), HC (119 mm, −3.192 SD), and FL (19 mm, −2.343 SD) were all below the expected values. At 21 weeks and 2 days, as well as at 26 weeks and 1 day of gestation, FGR persisted. HC, BPD, AC, and FL were all below the expected measurements for the respective gestational ages. At 26 weeks and 1 day of gestation, additional anomalies were identified, including a ventricular septal defect, an aberrant right subclavian artery, and bilateral renal hypoplasia. The left kidney measured 22 × 13 mm, while the right kidney measured 20 × 11 mm, with both kidneys exhibiting poorly defined outlines.

#### 3.1.3. Case 3

At 19 weeks and 6 days of gestation, FGR was identified, with the AC measuring 122 mm (−3.415 SD) and the EFW at 234 g (0.7th percentile). FGR persisted at 25 weeks and 4 days of gestation, at which AC was 170 mm (−4.227 SD), and the estimated weight was 513 g (0th percentile). Additionally, HC (205 mm, −3.688 SD) and BPD (54 mm, −3.106 SD) were below the expected values. Fetal MRI at 26 weeks and 4 days of gestation revealed that the fetal BPD (5.81 cm, −2.87 SD) was less than that corresponding to the gestational week, with no other cranial anomalies detected.

#### 3.1.4. Case 4

At 23 weeks and 6 days of gestation, ultrasound indicated FGR. AC measured 167 mm (−2.766 SD), and EFW was 456 g (0.4th percentile). At 27 weeks and 2 days of gestation, FGR persisted, with AC 196 mm (−3.033 SD), and the estimated weight was 720 g (0.1st percentile). At 28 weeks and 5 days of gestation, FGR continued, with AC 218 mm (−2.171 SD) and an estimated weight of 894 g (0.2nd percentile). Furthermore, BPD and HC below the expected range for gestational age were observed at 27 weeks and 2 days of gestation: HC (224 mm, −3.454 SD) and BPD (60 mm, −2.682 SD). This persisted at 28 weeks and 5 days of gestation: HC (245 mm, −2.489 SD) and BPD (64 mm, −2.605 SD).

#### 3.1.5. Case 5

At 23 weeks and 4 days of gestation, the fetus exhibited FGR. AC measured 163 mm (−2.908 SD), and EFW was 450 g (0.9th percentile). HC (191 mm, −2.682 SD) and BPD (51 mm, −2.11 SD) were below the expected values. Ultrasound at 31 weeks and 1 day of gestation revealed bilateral renal hypoplasia, with the left kidney measuring 28 × 14 mm and the right kidney measuring 29 × 14 mm. The kidneys demonstrated normal morphology, clear corticomedullary demarcation, and no apparent masses. Ultrasound at 31 weeks and 4 days indicated nonvisualization of the fetal gallbladder. By 34 weeks and 2 days, FGR persisted, with AC of 263 mm (−3.124 SD), an estimated weight of 1591 g (0.1st percentile), and both HC (278 mm, −3.124 SD) and BPD (75 mm, −2.889 SD) below the expected values.

#### 3.1.6. Case 6

At 21 weeks of gestation, the fetus exhibited FGR. AC measured 141 mm (−2.0 SD), and EFW was 304 g (0.3rd percentile). Both HC (164 mm, −2.021 SD) and BPD (38.6 mm, −3.789 SD) were below the expected values. FGR persisted at 23 weeks and 6 days of gestation, with AC measuring 168 mm (−2.2 SD) and the estimated weight at 441 g (0th percentile). HC (197 mm, −2.016 SD), BPD (51 mm, −2.597 SD), and FL (36 mm, −2.199 SD) were all below the expected values. Fetal cranial MRI at 27 weeks and 3 days of gestation showed that the fetal BPD (6.08 cm, −2.764 SD) was below the expected value, with no other cranial anomalies detected. The pregnant woman (Case 7) has a height of 140 cm, while her younger brother, mother, and father are 160, 140, and 160 cm, respectively. The pregnant woman holds a technical secondary school diploma and has been employed in a pharmacy.

### 3.2. Clinical Manifestations of Patients With RAUST

A total of 10 articles met the inclusion criteria, yielding a retrospective analysis of 39 cases, including six fetal cases and one adult case from our cohort (Supporting Information 1). Trio‐WES had been conducted on 34 patients and their parents, revealing de novo variants in 88.24% (30/34) of cases, while inherited variants from the affected parent were identified in 11.76% (4/34).

**Figure 2:**
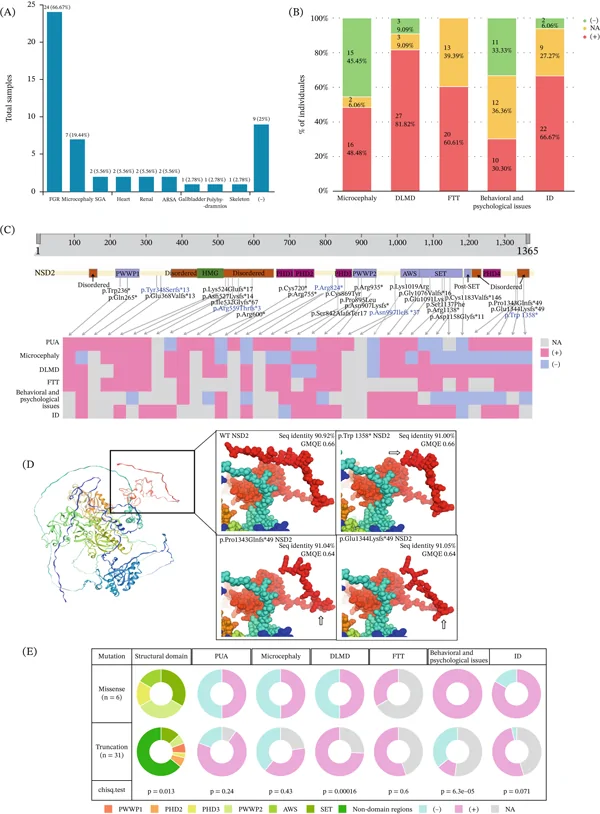
Phenotypic distribution of RAUST cases and NSD2 truncated mutant structure prediction models. (A) Prenatal ultrasound characteristics of the fetuses. ARSA: aberrant right subclavian artery; FGR: fetal growth restriction; SGA: small for gestational age infant; Heart: heart malformations (including ventricular septal defect and interruption of the aortic arch); Renal: bilateral renal hypoplasia; Gallbladder: nonvisualization of the gallbladder; Skeleton: flexion contractures of the feet, knees, and elbows. (B) Postpartum phenotypic characteristics of RAUST cases. DLMD: developmental language and motor delay; FTT: failure to thrive; ID: intellectual disability. (C) NSD2 variants in 37 patients with RAUST. Cases from our institution are highlighted in blue. The various structural domains of NSD2, including proline‐tryptophan‐tryptophan‐proline domain (PWWP), the zinc‐finger domain of the Plant‐HomeoDomain type (PHD), high mobility group box domain (HMG), and the catalytic methyltransferase domain (MTD, comprising the AWS, SET, and post‐SET domains), are annotated based on the UniProt database (https://www.uniprot.org/). PUA: prenatal ultrasound abnormalities. Grey: “NA” indicates that no data is available; pink: (+) indicates that the patient has this phenotype; blue: (−) indicates that the patient does not have this phenotype. (D) 3D structure prediction model of wild‐type and truncated NSD2 proteins. This figure presents the 3D structure prediction model of the wild‐type NSD2 protein alongside schematic representations of the structural changes in the NSD2 truncated mutant proteins p.Trp1358∗, p.Pro1343Glnfs∗49, and p.Glu1344Lysfs∗49. The arrows in the figures indicate the locations where the peptide begins to change. These predictions were generated using Swiss‐Model. (E) Donut chart and chi‐square test of phenotype and NSD2 variant in 37 patients with RAUST. The *p* value indicating significance is presented in the final row of the chi‐square test output. The donut chart visually represents the proportion of various phenotypes associated with each variant type, while the chi‐square test *p* values indicate the statistical significance of the relationship between variant type and clinical phenotype.

Of the 39 cases, prenatal records were unavailable for three cases. Figure 2A illustrates that among the 36 fetuses with documented prenatal phenotypes, 75% (27/36) exhibited abnormal prenatal ultrasound findings. The most common ultrasound anomaly was FGR, followed by microcephaly, SGA, cardiac anomalies, bilateral renal hypoplasia, and an aberrant right subclavian artery. Among the cardiac anomalies, two cases presented with ventricular septal defects, and one case involved an interrupted aortic arch. Other phenotypes, each observed in a single case, included gallbladder nonvisualization, flexion contractures of the feet, knees, and elbows, and polyhydramnios.

Postnatally, abnormal sonograms were observed in 29.63% (8/27) of children with available outcome data. Genitourinary anomalies were the most common, reported in 25.93% (7/27) of cases, including three cases of cryptorchidism, two of renal hypoplasia, and single cases of Stage II chronic kidney disease, right kidney rotation, left hydronephrosis, hypospadias, and shawl scrotum. Cardiovascular anomalies followed, accounting for 11.11% (3/27), with one case each of mild left pulmonary artery peripheral stenosis, small nonhemodynamically significant patent foramen ovale, and mild mitral valve prolapse. One case also showed hepatomegaly on ultrasound.

Figure 2B demonstrates that the most prevalent postnatal clinical manifestation was speech‐motor delay (84.38%, 27/32), followed by intellectual disability (68.75%, 22/32) and physical delay (62.50%, 20/32). Furthermore, microcephaly and behavioral and psychological issues were observed at high rates.

Among our 39 cases, two had unknown variant types and specific variant loci. Of the remaining 37 cases with known variant sites, variants were found in 5.41%, 5.41%, 2.70%, 13.51%, 2.70%, 16.22%, and 54.05% of the cases in the PWWP1, PHD2, PHD3, PWWP2, AWS, SET, and nonstructural domains of the NSD2 protein, respectively. Combining Figure 2C and Figure 2E, we can see that variants occurring in the nonstructural domain accounted for the largest proportion, and all were truncation variants, which directly impact the integrity of peptide chain. Furthermore, we found that truncation in the peptide chain behind the last domain PHD4 has pathogenic effects. As shown in Figure 2D, the peptide chain at the protein’s C‐terminus is significantly shortened. Although this does not directly alter the composition of the domain, it may influence the spatial conformation of the NSD2 protein and its interaction with histone H3.

Table 2 indicated a statistically significant difference in the presence or absence of behavioral and psychological issues based on variant location—variants within structural domains were associated with a higher prevalence of such issues. Notably, variants located between different structural domains had no statistically significant difference in behavioral and psychological issues among these patients (*p* = 1).

**Table 2:** Fisher’s exact test evaluating the association between NSD2 variant location (within structural domains) and variant type (missense/truncation) with phenotypes.

P (Fisher’s)	PUA	Microcephaly	DLMD	Behavioral and psychological issues	ID
Structural domain (+)/(‐)	0.264	0.725	0.113	0.008 ^∗^ (diff. 1)	1
Variant (missense/truncation)	0.306	1	0.005 ^∗^	0.004 ^∗^	0.462

Abbreviations: DLMD: developmental language and motor delay; ID: intellectual disability; PUA: prenatal ultrasound abnormalities.

^∗^The *p* value is statistically significant; diff.: Fisher’s exact test to assess the association between variants located in different structural domains and phenotypes.

Among the 37 cases with known specific variant types, 16.22% (6/37) were missense, while 83.78% were truncating variants. The six pathogenic missense variants, depicted in Figure 2E, were distributed across three distinct structural domains of NSD2: the PHD3 zinc finger, PWWP2, and the catalytic methyltransferase domain, which comprises three subdomains (AWS, SET, and post‐SET). As outlined in Table 2 and Figure 2E, a statistically significant difference was observed between variant types and the presence or absence of speech‐motor delay and behavioral–psychological issues. Truncating variants were associated with a higher incidence of speech–motor delay. Notably, all missense variants were correlated with behavioral and psychological issues; in contrast, only 26.67% of patients with truncating variants exhibited behavioral and psychological symptoms. Patients with truncating variants also demonstrated a relatively higher prevalence of prenatal ultrasound abnormalities and intellectual disabilities, although the difference was not statistically significant.

### 3.3. Spatiotemporal Expression of *NSD2*

**Figure 3:**
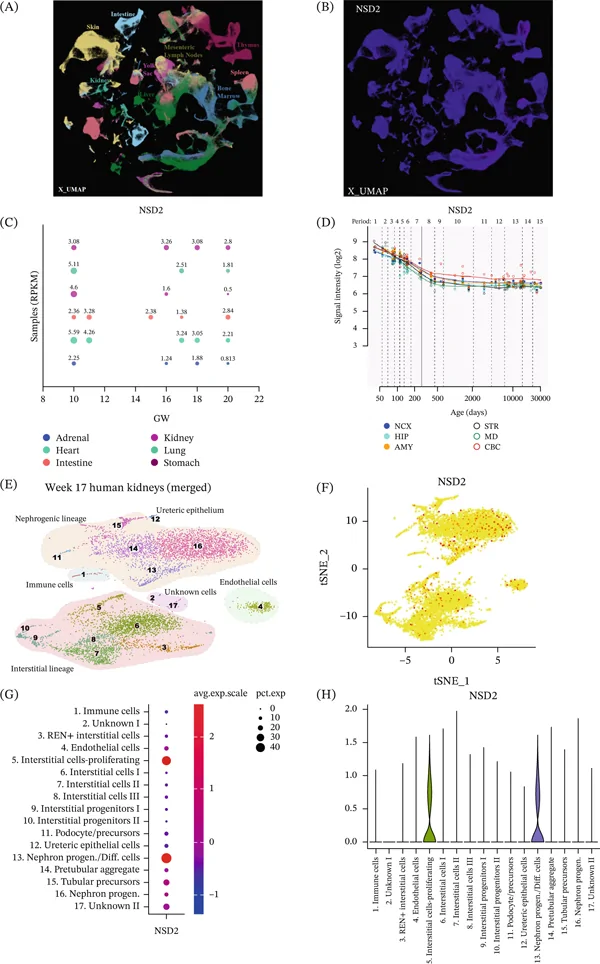
Single‐cell transcriptomics and gene expression atlases of *NSD2* in multi‐source public databases. (A–B) UMAP dimensionality reduction plots of single‐cell RNA‐seq data from nine human developmental tissues (fetal bone marrow, fetal intestine, fetal kidney, fetal liver, fetal mesenteric lymph nodes, fetal skin, fetal spleen, fetal thymus, and yolk sac) during gestational Weeks 4–17, based on the HDCA database (A), and the UMAP plot of *NSD2* expression across these tissues (B), illustrate tissue‐specific expression patterns of *NSD2*. Panel A: The color‐coded clusters represent single‐cell distributions following UMAP dimensionality reduction (each point corresponds to one cell; colors denote tissue‐specific subpopulations). Panel B: Gene expression levels are indicated by a color gradient (red = high expression; blue = low expression). (C) Scatter plot depicting *NSD2*‐associated circular RNA expression from the NCBI database. The *x* axis represents gestational age (8–22 weeks), and the *y* axis displays RPKM values. Data points are color‐coded by human tissue origin (stomach, heart, lung, kidney, intestine, and adrenal), revealing spatiotemporal expression dynamics of circular RNAs. (D) Line graph illustrating *NSD2* expression levels across distinct brain regions at various human fetal developmental stages, derived from the HBT database. The *x* axis denotes developmental time (embryonic to postnatal days), and the *y* axis shows log‐transformed signal intensity for gene expression quantification. Markers are differentiated by color and shape to represent brain regions (NCX: neocortex; STR: striatum; HIP: hippocampus; MD: mediodorsal thalamic nucleus; AMY: amygdala; CBC: cerebellum). (E–H) Single‐cell sequencing analysis of human fetal kidney at 17 weeks of gestation (RBK database). (E) Clustering visualization of single‐cell data. (F) t‐SNE plot highlighting *NSD2* expression patterns. (G) Scatter plot displaying *NSD2* expression across kidney cell types. (H) Violin plot displaying *NSD2* expression across kidney cell types, highlighting proliferative stromal cells and renal progenitor/differentiated cells.

HDCA data indicate that *NSD2* is highly expressed in human hematopoietic and immune system‐related tissues (e.g., fetal thymus, bone marrow, and liver) compared with other tissues (Figure 3A,B). The scatter plot from the NCBI database demonstrates that *NSD2*‐associated circular RNAs exhibit a tissue‐specific expression pattern in human fetal tissues between 8 and 22 weeks, indicating that *NSD2* may play an important role in the early development of the embryonic heart, lung, and kidney (Figure 3C). HBT data show that *NSD2* is highly expressed in various human brain regions (including the neocortex, striatum, thalamus, amygdala, hippocampus, and cerebellar cortex) from early embryonic stages to post‐birth, with notably high expression levels during early development (Figure 3D). Data from the RBK database regarding human kidneys at 17 weeks of gestation indicate that *NSD2* is highly expressed in proliferative stromal cells and renal progenitor cells (Figure 3E–H), suggesting its potential involvement in the construction of renal stroma and the differentiation of renal units.

## 4. Discussion

In this study, we systematically analyzed the prenatal–postnatal phenotypic spectrum of RAUST and the corresponding genotypes in *NSD2* gene by starting with six prenatal cases and combining historical literature. Through detailed analysis of genotype–phenotype correlation, some suggestive clues for early disease diagnosis were obtained.

The most prevalent postnatal phenotypes were delayed speech and motor development, followed by intellectual disability and physical developmental delay, which are similar to previous studies [3].

The most common prenatal phenotype of RAUST is FGR, but there is significant variation in the onset time and severity. FGR is characterized by the fetus’s failure to achieve its genetically predetermined growth potential. FGR is diagnosed when ultrasound measurements—such as EFW or AC—fall below the 10th percentile for the corresponding gestational age. When a fetus has only SGA, invasive prenatal diagnosis cannot be triggered [16, 17]. In previous articles, more than half (18 out of 30) of the cases had FGR, but none underwent timely invasive prenatal testing. Additionally, the most common prenatal phenotype aligns with the typical manifestations of postnatal growth retardation. This suggests that the effects of *NSD2* gene on physical development are persistent. Case 1 supports this observation, where FGR improved in the later stages of pregnancy, but the child still exhibited physical developmental delays after birth.

The second most common prenatal ultrasound abnormality is microcephaly. In this cohort, prenatal ultrasounds identified microcephaly in all six affected fetuses, while only one case had been reported in the literature [3]. One possible reason is that all the cases in the literature are postnatal cases, and the methods of HC measurement differ between prenatal and postnatal periods [18, 19]. The prenatal data of those postnatal cases in the literature are unavailable. Therefore, this indicator still requires research on a larger number of both prenatal and postnatal cases. Additionally, prenatal ultrasound abnormalities included cardiac malformations and renal hypoplasia, which were the most common postnatal ultrasound abnormalities. This suggests that cardiac malformations and bilateral renal hypoplasia might serve as potential prenatal ultrasound markers for RAUST.

Notably, the mother of Case 6 (Case 7) carried the same *NSD2* variant. She was 140 cm height with a college education and pharmacy work experience. Her family showed mild short stature, but no genetic testing or formal intellectual assessment was performed. Together with previous literature and Figure 2C, these findings support that RAUST‐related variants exhibit incomplete penetrance and variable expressivity, broadening the clinical spectrum of this disorder.

To date, comprehensive analyses of the correlation between *NSD2* variants and their corresponding phenotypes are lacking. Among more than 40 identified *NSD2* variants, missense and truncating variants are distributed throughout the entire gene [6]. Zanoni et al. suggested that, compared with patients with truncating variants, those with missense variants present milder physical growth retardation but exhibit more frequent behavioral and psychological issues [3].

Among the 37 cases with identified variants, six involved missense variants, and 31 involved truncating variants. Zanoni’s team performed structural analyses and in vitro methylation assays on these six pathogenic missense variants, indicating that all missense variants in the structural domains disrupt NSD2 folding and function, impairing its ability to generate H3K36me2 [3]. Multiple research groups have demonstrated that loss of NSD2 function significantly reduces H3K36 dimethylation, based on cellular and in vitro functional assays, as well as Western blot and immunofluorescence analyses [3, 20]. These findings suggest that H3K36me2 may function as a molecular marker in the diagnosis of RAUST. Figure 1B illustrates that the severity of growth parameter impairment correlates with the proximity of truncating variants to the N‐terminal region. In Case 4, there is a full‐length deletion of the *NSD2* gene; however, the phenotype is less severe than that in cases with partial deletions. The specific mechanism remains unclear and requires further investigation. Figure 2C demonstrates that truncating variants nearer to the N‐terminus are associated with a higher rate of positive phenotypes. These findings indicate that the severity of phenotypes is closely associated with the structural integrity, domain composition, and spatial conformation of the NSD2 protein.

NSD2 contains key domains: SET (catalyzes H3K36me2 methylation) [21], PWWP (binds H3K36 nucleosomes) [22], PHD zinc finger (preserves catalytic activity, interacts with methylated histones) [21], and HMG box (aids localization) [23–25]. The interaction pattern between the NSD2 protein and nucleosome H3 can be influenced by structural changes in NSD2 [26]. It is currently known that PHD4 is the last functionally important domain of the NSD2 protein; however, existing prenatal (Case 1) and postnatal cases [3, 6, 27] have shown that truncating variants downstream of the PHD4 domain are still pathogenic with severe phenotypes. We acknowledge that the full‐length NSD2 model presented herein is based on a computationally predicted mouse template (AlphaFold) rather than an experimentally determined structure. As such, this model should be regarded as hypothetical, and all structural interpretations derived from it require experimental validation, such as cryo‐electron microscopy (cryo‐EM) or functional assays.

The database revealed the important role of *NSD2* in the development of multiple organs. First, the developmental regulation of circular RNAs may be evolutionarily conserved; the dynamic expression patterns of circular RNAs in the NCBI database support *NSD2*’s potential involvement in multi‐organ development through noncoding RNA regulatory networks [15]. Second, HBT data reveal high *NSD2* expression in early embryonic brain regions, indicating its potential importance for nervous system development [28, 29]. Meanwhile, kidney‐specific expression data from the RBK database directly demonstrate *NSD2*’s functional specificity in renal stroma formation and nephron differentiation, suggesting a potential molecular mechanism underlying congenital kidney developmental abnormalities [30–32]. Furthermore, the HDCA data are consistent with previous literature, confirming *NSD2*’s involvement in fetal hematopoietic and immune system development—particularly through its regulation of multiple hematopoietic stages, B‐ and T‐cell differentiation, which correlates with the characteristic immune deficiency phenotypes observed in patients with WHS [33, 34]. However, clinical observations indicate that only a minority of patients experience recurrent infections, suggesting that the relationship between phenotype and genotype of *NSD2* needs further study. In summary, the conservation of circular RNA‐mediated developmental regulation provides a potential molecular context for *NSD2*’s role in multi‐organ development. Multidimensional data from HBT, RBK, and HDCA collectively highlight *NSD2*’s important role in the development of the nervous, renal, and hematopoietic‐immune systems, with its dysfunction potentially serving as a molecular trigger for congenital developmental abnormalities.

In this study, we expanded the phenotypic spectrum of RAUST to a prenatal context, supplemented knowledge regarding the early diagnosis of affected fetuses, and established a comprehensive analytical model for examining associations between *NSD2* variant and RAUST synopsis. However, the study has a notable limitation: the small number of cases, particularly fetal cases. Furthermore, for the diagnosis of RAUST, in addition to genetic testing for *NSD2*, the measurement of H3K36me2 levels can also aid in diagnosis, particularly when a variation of uncertain significance in *NSD2* is found. Therefore, further research is required to advance early diagnosis approaches and elucidate the pathogenic mechanisms underlying RAUST.

In conclusion, the fetal phenotype of RAUST exhibits significant variability. The most common prenatal manifestation of fetal RAUST is FGR or SGA. When a fetus presents FGR or SGA accompanied by microcephaly, cardiac malformations, or renal abnormalities, genetic testing should be recommended for the fetus to facilitate early prenatal diagnosis and optimize clinical management.

## Author Contributions

Conceptualization: Yan Zhang. Data curation: Yi Kong, Xingwang Wang, Yousheng Wang. Formal analysis: Yi Kong, Xingwang Wang, Li Guo. Project administration: Yi Kong, Yousheng Wang. Funding acquisition: Yan Zhang. Supervision: Yan Zhang. Validation: Jianhong Chen, Yan Ji, Jiaolan Liu. Visualization: Wanfang Xu, Weiling Du, Haiyan Wang. Writing – original draft: Yi Kong, Yan Zhang. Writing – review and editing: All authors.

## Funding

This study was supported by National Key Research and Development Program of China, 10.13039/501100012166, No. 2024YFC2707000, 2024YFC2707001.

## Ethics Statement

The published clinical information obtained written informed consent from the pregnant women themselves and was approved by the ethics committee of our hospital.

## Conflicts of Interest

The authors declare no conflicts of interest.

## Supporting information


**Supporting Information** Additional supporting information can be found online in the Supporting Information section. S1 Supporting Information 1 (Sheet 1 contains detailed information regarding the genes and phenotypes of the patients included in this study, while Sheet 2 provides specific growth parameter values at various gestational weeks for the six RAUST fetuses in our hospital.)

## Data Availability

Data available in article supporting information.
